# The Protective Effect of Sodium Ferulate and Oxymatrine Combination on Paraquat-induced Lung Injury

**Published:** 2015

**Authors:** Wei Wang, Xiaokun Pei, Mengxin Xu, Songmei Sun, Chunlei Zhang, Keying Mu, Zhifeng Liu

**Affiliations:** School of Pharmacy, Yantai University, No. 30, Qingquan Road, Laishan District, Yantai, Shandong, China.

**Keywords:** Sodium ferulate, Oxymatrine, Paraquat, Lung, Inflammation

## Abstract

Experimental evidence suggested that sodium ferulate (SF) and oxymatrine (OMT) combination had synergistic anti-inflammatory and antioxidant effects. We hypothesized that SF and OMT combination treatment might have protective effects on paraquat-induced acute lung injury. In our study, the Swiss mice were randomly divided into seven groups, including control, paraquat (PQ), SF (6.2 mg/Kg/day); OMT (13.8 mg/Kg/day) and three SF+OMT groups (3.1 + 6.9; 6.2 + 13.8 and 12.3 + 27.7 mg/Kg/day). The mortality and death time were monitored. Sprague-Dawley rats were randomly divided into seven groups including control, PQ, SF (3.1 mg/Kg/day); OMT (6.9 mg/Kg/day) and three SF+OMT groups (1.6 + 3.4; 3.1 + 6.9 and 6.2 + 13.8 mg/Kg/day). The lung wet/dry weight (W/D) ratio, lung histopathologic changes, C-reactive protein (CRP), interleukin-6 (IL-6), nuclear factor κB (NF-κB), malondialdehyde (MDA) and superoxidase dismutase (SOD) were analysed. Compared with PQ group, the mortality significantly decreased and the death time prolonged in SF and OMT combination treatment groups of mice. Also in SF and OMT combination treatment groups of rats, the increased lung W/D ratio and histopathological score induced by PQ injection were significantly decreased; the levels of CRP, IL-6, NF-κB and MDA in serum and lung homogenate were significantly decreased; the SOD activities in serum and lung homogenate were improved. These results suggested that SF and OMT combination had an obvious protective effect on PQ-induced lung injury. The anti-inﬂammatory and antioxidant effect might be involved in the mechanism.

## Introduction

It is well known that the lung is one of the friable organs which can be injured by many factors, such as virus, bacteria, Lipopolysaccharide (LPS) and some chemical substances. Paraquat (1,1’-dimethyl-4,4’-bipyridinium dichloride, PQ) is a nonselective herbicide widely used for its high efficiency and low residues in agriculture worldwide. PQ intoxication, after intentional or accidental ingestion, usually led to lung injury (1, 2) and importantly, the death was mainly caused by irreversible respiratory failure (3, 4). It was reported that the mortality of PQ intoxication patients was as high as 55.2% (5). Many researchers (6, 7) believed that the production of superoxide radicals and its triggered oxidative damage, oxidative stress-related cell damage, and followed inﬂammatory reaction were mainly associated with the mechanisms of PQ-induced lung injury. The pathophysiologic progress including inﬂammatory inﬁltration into the alveolar spaces, excessive collagen deposition and proliferation of the pulmonary edema fluid ultimately lead to pulmonary ﬁbrosis and respiratory failure. Now, some anti-inflammatory and antioxidant drugs had been demonstrated curative effects for PQ intoxication, such as dexamethasone and methylprednisolone which could reduce the inflammatory reaction, including reducing pulmonary vascular permeability and pulmonary fibrosis (8, 9). 

In our previous studies, we found that sodium ferulate (SF) and oxymatrine (OMT) combination had remarkable synergetic anti-inﬂammatory and antioxidant effects (10). So we hypothesized that SF and OMT combination treatment might alleviate PQ-induced lung injury. In this study, the mice and rats PQ intoxication models were duplicated to observe the protective effect of SF and OMT combination, and it’s anti-inflammatory and antioxidant mechanism were also investigated. We hoped these data would provide experimental evidence in a new lung injury protection drug development.

## Experimental

*Drugs and chemicals*

SF (molecular formula, C_10_H_9_NaO_4_·2H_2_O; molecular weight, 252.20; CAS, 24276-84-4; HPLC purity > 99%), OMT (molecular formula, C_15_H_24_N_2_O_2_·H_2_O; molecular weight, 282.38; CAS, 16837-52-8; HPLC purity > 98%) were provided by Beijing SL Pharmaceutical Co., *Ltd*. (Beijing, China). PQ was produced by Shandong Green Abundant Pesticide Co., (Weifang, China). MDA and SOD kits were produced by Jiancheng Bioengineering Institute (Nanjing, China). Protein test kits were produced by Shanghai Beyotime Institute of Biotechnology. Enzyme linked immunosorbent Assay (ELISA) kits for determination of IL-6, CRP and NF-κB were produced by Groundwork Biotechnology Diagnosticate Ltd. (San Diego, CA, USA) and imported by Yantai Dite Trade Co., Ltd. (Yantai, China).

*Animals*

Swiss mice (18–20 g) and Sprague–Dawley rats (120–150 g) were purchased from the Shandong Luye Pharmaceutical Co., *Ltd* (Quality Certiﬁcated Number: Lu 20090013). The animals were kept under standard conditions (temperature, 23 ± 2 °C; humidity, 55 ± 5%, 12 h light/dark cycle) and acclimatized to the laboratory environment for 3-7 days. Only water was provided to the animals within 12 h before the experiment and then the animals were fed regularly after the PQ injection. Experimental procedures in this study were approved by the Experimental Animal Management Center of Yantai University and performed in accordance with the Guidelines for the Care and Use of Laboratory Animals. The duration of the experiments was as short as possible.

*Mortality and death time* *of* *mice*

The animals were randomly divided into seven groups: (1) control (saline), (2) PQ (saline), (3) SF (6.2 mg/Kg/day), (4) OMT (13.8 mg/Kg/day), (5) SF+OMT (low dose group, 3.1 + 6.9 mg/Kg/day), (6) SF+OMT (middle dose group, 6.2 + 13.8 mg/Kg/day), (7) SF+OMT (high dose group, 12.3 + 27.7 mg/Kg/day). There were ten animals in each group, male and female in half. Optimal molar ratio of SF and OMT (1:2) combination was obtained from the tests of pharmacology and pharmaceutical. We found that when the molar ratio of SF and OMT was 1:2, the combination had the best pharmacological activity and the system was the most stable with pH value of 7.0 (will be published in another paper). PQ (20 mg/Kg) was administered to the animals by intraperitoneal injection except the animals in control group (saline was injected). Thirty minutes later, saline or corresponding drug was hypodermic injected, and followed two times administration daily, respectively at 8:00 AM and 4:00 PM until the third day. The animals were observed carefully at the first 8 hours and every four hour observation was followed until 52 hours after PQ administration. The death time was recorded as the following principle: if the animal died during 8 hours to 12 hours period, the death time was recorded as 12 hours, and so on. If the animal did not die at 52 hours point, the death time was recorded as 52 hours.

*L* *ung* *injury* *model in rats* *and drug administration*

The rat PQ-induced lung injury model was duplicated referring to previous report (11). In our preliminary studies, we found that with the dose of 6.2 + 13.8 mg/Kg/day, SF and OMT combination treatment showed curative effect on PQ intoxication rats. So, we designed seven groups as following: (1) control (saline), (2) PQ (saline), (3) SF (3.1 mg/Kg/day), (4) OMT (6.9 mg/Kg/day), (5) SF+OMT (low dose group, 1.6 + 3.4 mg/Kg/day), (6) SF+OMT (middle dose group, 3.1 + 6.9 mg/Kg/day), (7) SF+OMT (high dose group, 6.2 + 13.8 mg/Kg/day). There were ten animals in each group, male and female in half. Except the animals in control group (saline was administered), all other animals were administered PQ (25 mg/Kg) by intraperitoneal injection, and 30 minutes later, the drug or saline was administered to the animals through hypodermic injection, and two times injections daily at 8:00 AM and 4:00 PM respectively until the third day.

*Preparation of serum and tissue homogenates* *of* *rats*

Preliminary experiment showed that the rats began to die after 60 hours of PQ injection. So, on the third day, about 56 hours later after PQ injection (when no rats died), the animal was anesthetized with  diethyl ether and 5 mL blood was drawn via abdominal aortic. The blood samples were centrifuged at 2500 rpm for 10 minutes at 4 ^o^C. The serum was separated and stored at −80 ^o^C for biochemical analysis. Then the rat was killed and the lung was rapidly excised. The lower lobe of right lung (n = 10) was fixed by formaldehyde for histopathological analysis, and the upper lobe (n = 10) was homogenized in buffer solution, prepared for 10% lung homogenate with Vertishear tissue homogenizer (Virtis, Gardiner, NY, USA). The supernatant was stored at −80 ^o^C for further biochemical analysis. The left lung was used to evaluate lung W/D ratio as following.

*Lung W/D ratio* *of* *rats*

After the animal was killed and lung was excised as mentioned above, the left lung (n = 10) was weighed and then dried in a drying oven at 60 °C for 72 h and weighed again as Oliveira described (12). The lung W/D ratio represented the degree of lung edema was calculated using follow formula: 

W/D ratio = wet weight/dry weight

*Biochemical analysis*

CRP, IL-6 and NF-κB levels in serum and lung homogenate were measured with ELISA kits according to the manufacturers’ manual. MDA level and SOD activity in serum and lung homogenate were assayed using MDA and SOD kits as described previously (13, 14). In brief, MDA level was measured based on the thiobarbituric acid method with a maximal absorbance at 532 nm, and SOD activity was measured on the basis of SOD-mediated inhibition of nitrite formation from hydroxyammonium in the presence of superoxide radical anion (O^2−^) generators (xanthine/xanthine oxidase system).

*Histopathological analysis*

The animal's right lower lobe (n = 10) was fixed with 10% formalin for histopathological specimen. The specimens were embedded in paraffin, sectioned and stained with eosin and hematoxylin. As the report of Yuan *et al*. (15), the degree of inflammatory cell infiltration situation and lung injury were scored under light microscopy from 0–4 as follows: 0 (no damage); 1 (slight diffuse damage of neutrophilic in alveolar walls, no thickening of alveolar walls, no hemorrhage, < 25 % congestion of alveolar space); 2 (diffuse damage of mononuclear and neutrophilic in alveolar walls and slight thickening of the alveolar walls, at least ﬁve erythrocytes per alveolus in one to ﬁve alveoli, < 25-30 % congestion of alveolar space,); 3 (two or three times thickening of the alveolar walls, at least ﬁve erythrocytes in ﬁve to ten alveoli, 30-50 % congestion of alveolar space); 4 (alveolar wall thickening with up to 50% of lung consolidated, at least ﬁve erythrocytes in more than ten alveoli, > 60 % congestion of alveolar space). The validity of the method for evaluation of the lung injury had been well established and the tissue sections were evaluated by the independent blinded observer Dr. Yujun Li, Director of Pathological Department, Shandong Luye Drug Safety Evaluation Center (Yantai, Shandong Province; Good Laboratory Practice Lab. certiﬁed by State Food and Drug Administration of China).

*Statistical analysis*

The results were expressed as means ± SEM and data were analysed by one-way ANOVA, with the Statistical Product and Service Solutions (SPSS 17.0, USA). The differences of the mortality between two groups was analysed by the Chi-squared test (X^2^ test). A value of *P* < 0.05 was taken as statistical signiﬁcant (*P < 0.05; **P < 0.01)**.**

## Results

*Effects of SF and OMT combination* *or the drug used alone on mortality and death time of mice*

**Figure 1:**
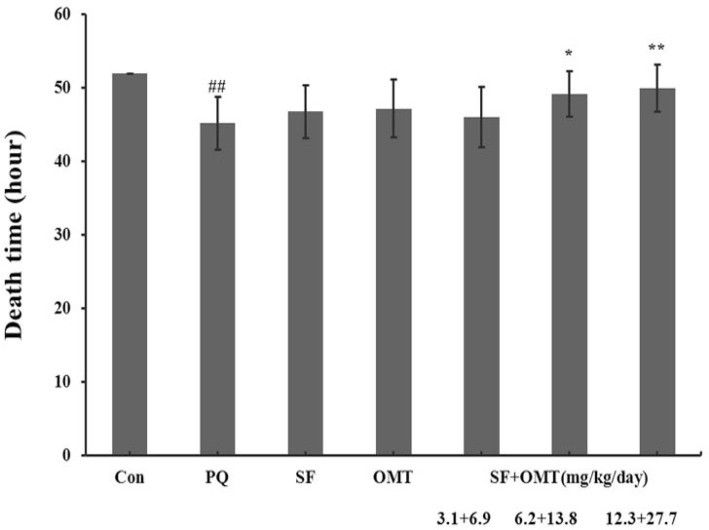
Effects of SF and OMT combination on the death time of PQ-intoxication mice. CON, PQ, SF and OMT represent the control group, paraquat group, sodium ferulate group (6.2 mg/Kg/day) and oxymatrine group (13.8 mg/Kg/day), respectively. SF+OMT represents sodium ferulate and oxymatrine combination treatment groups, and the 3.1 + 6.9, 6.2 + 13.8 and 12.3 + 27.7 respectively represent drug administration doses of SF+OMT in the low dose group, middle dose group and high dose group. Data are expressed as means ± SEM. n = 10 in each group. ^##^*P* < 0.01 compared with the control group. ^*^P < 0.05, **P < 0.01 compared with the PQ group

After PQ injection, all animals died within 52 hours in PQ group and no animal died in control group. Compared with the PQ group, treatment with SF and OMT combination could decrease the mortality (Table 1) and prolonged the death time (Figure 1). As showed in the Table 1, the mice mortality was 80%, 50%, 30% at 48 hours, and 90%, 80%, 60% at 52 hours respectively in low dose group, middle dose group and high dose group of the combination treatment. Compared with the PQ group, treatment with either SF (6.2 mg/Kg/day) or OMT (13.8 mg/Kg/day) alone could not significantly decrease the mortality (100% and 90% respectively at 52 hours), or prolong the death time.

**Table 1:** Effects of the combination of SF and OMT on the mortality of PQ-intoxication mice.

**Group**	**Dose ** **(mg/Kg/day)**	**Mice** **number**	**Mortality within 52 h**	
			** 48 h**	**52 h**
Con	--	10	0%	0%
PQ	--	10	90%##	100% ##
SF	6.2	10	80%	100%
OMT	13.8	10	70%	90%
SF+OMT (low dose)	3.1+6.9	10	80%	90%
SF+OMT(middle dose)	6.2+13.8	10	50%	80%
SF+OMT(high dose)	12.3+27.7	10	30%**	60%^*^

CON, PQ, SF and OMT represented the control group, PQ group, SF group and OMT group, respectively. SF + OMT represented the group of the combination of SF and OMT. The statistical different between two groups was analysed by X^2^ test.

## *P *< 0.01 versus the control group.

* *P *< 0.05,

** *P *< 0.01 versus the PQ group.

*Effects* *of SF and OMT combination* *or the drug used alone on lung W/D ratio and histopathological changes of rats*

**Figure 2:**
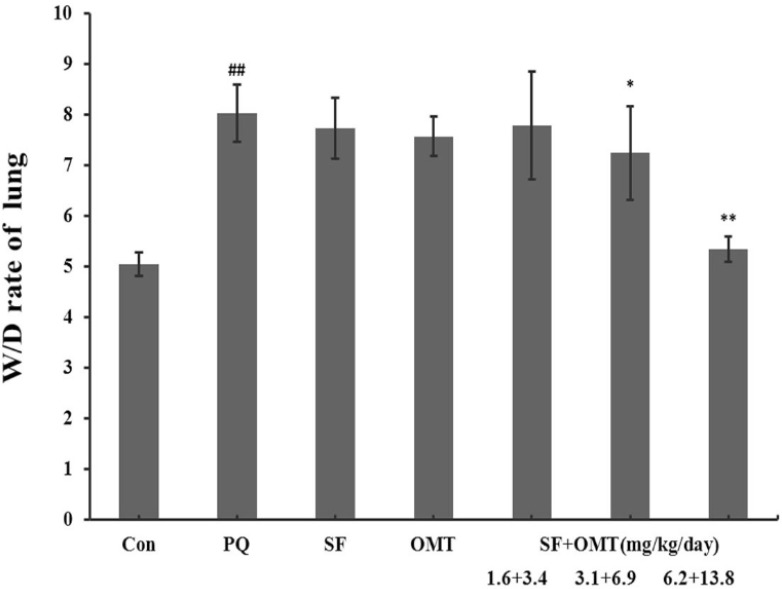
Effects of SF and OMT combination on the lung Wet/Dry (W/D) ratio of PQ intoxication rats. CON, PQ, SF and OMT represent the control group, paraquat group, sodium ferulate group (3.1 mg/Kg/day) and oxymatrine group (6.9 mg/Kg/day), respectively. SF+OMT represents sodium ferulate and oxymatrine combination treatment groups, and the 1.6 + 3.4, 3.1 + 6.9 and 6.2 + 13.8 respectively represent drug administration doses of SF+OMT in the low dose group, middle dose group and high dose group. The lung W/D ratio was determined at 56 h after the PQ injection. Data are expressed as means ± SEM. n = 10 in each group. ^##^*P* < 0.01 compared with the control group. ^*^P < 0.05, **P < 0.01 compared with the PQ group

As shown in Figure 2, the lung W/D ratio increased markedly in PQ group. In the middle dose group and high dose group of the combination treatment (SF+OMT 3.1 + 6.9, 6.2 + 13.8 mg/Kg/day), the lung W/D ratio was significantly decreased compared with the PQ group. Treatment with SF (3.1 mg/Kg/day) or OMT (6.9 mg/Kg/day) alone could not significantly decrease the lung W/D ratio.

**Figure 3:**
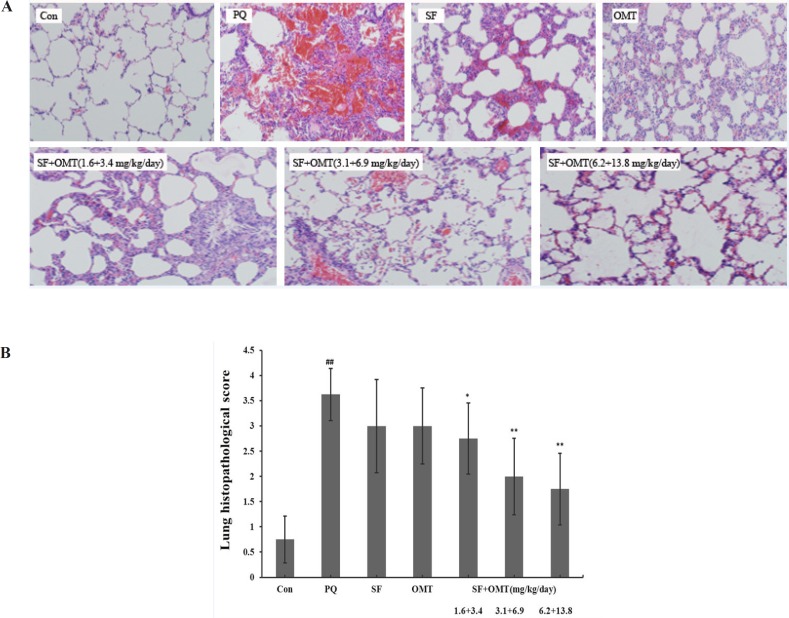
Effects of SF and OMT combination on the lung histopathological changes of PQ-intoxication rats. CON, PQ, SF and OMT represent the control group, paraquat group, sodium ferulate group (3.1 mg/Kg/day) and oxymatrine group (6.9 mg/Kg day), respectively. SF+OMT represents sodium ferulate and oxymatrine combination treatment groups, and the 1.6 + 3.4, 3.1 + 6.9 and 6.2 + 13.8 respectively represent drug administration doses of SF+OMT in the low dose group, middle dose group and high dose group. The degree of lung injury was scored under light microscopy from 0–4 as follow: 0 (no damage), 1 (slight diffuse damage of neutrophilic in alveolar walls, no thickening of alveolar walls, no hemorrhage, < 25 % congestion of alveolar space); 2 (diffuse damage of mononuclear and neutrophilic in alveolar walls and slight thickening of the alveolar walls, at least ﬁve erythrocytes per alveolus in one to ﬁve alveoli, < 25-30 % congestion of alveolar space,); 3 (two or three times thickening of the alveolar walls, at least ﬁve erythrocytes in ﬁve to ten alveoli, 30-50% congestion of alveolar space); 4 (alveolar wall thickening with up to 50% of lung consolidated, at least ﬁve erythrocytes in more than ten alveoli, > 60 % congestion of alveolar space). Data are expressed as means ± SEM. n = 10 in each group. ^##^*P* < 0.01 compared with the control group. *P < 0.05, **P < 0.01 compared with the PQ group.

As shown in Figure 3a, the lung tissue injury or inflammation changes were almost not observed in the control group. In PQ group, light microscope showed obvious pulmonary edema sign and infiltration of lots of inflammatory cell and erythrocyte. The decreased inflammtion infiltration was observed in the SF and OMT combination treatment groups, and the effects were much stronger than the drug used alone, which were scored in Figure 3b.

*Effects of* *SF and OMT combination or the drug* *used alone on the levels of CRP, IL-6, NF-κB in serum induced by PQ* *of* *rats*

**Figure 4:**
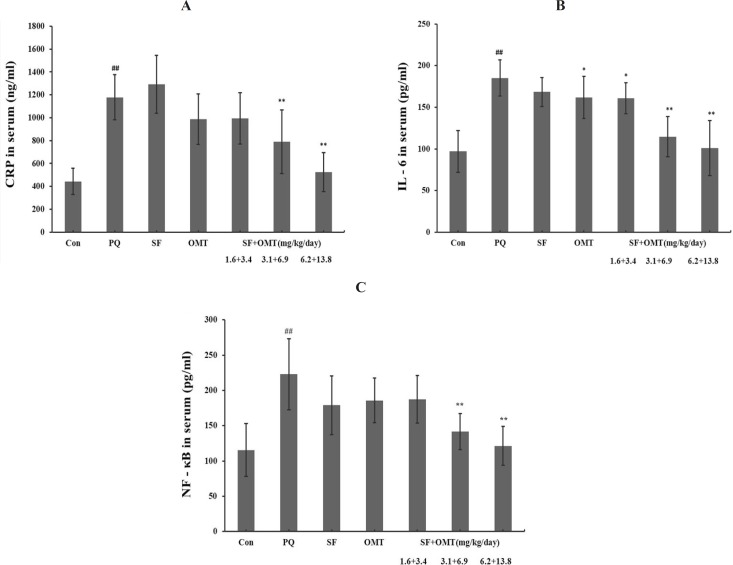
Effects of SF and OMT combination on the levels of CRP (a), IL-6 (b), NF-κB (c) in the serum of the PQ-intoxication rats. CON, PQ, SF and OMT represent the control group, paraquat group, sodium ferulate group (3.1 mg/Kg/day) and oxymatrine group (6.9 mg/Kg/day), respectively. SF+OMT represents sodium ferulate and oxymatrine combination treatment groups, and the 1.6 + 3.4, 3.1 + 6.9 and 6.2 + 13.8 respectively represent drug administration doses of SF+OMT in the low dose group, middle dose group and high dose group. Data are expressed as means ± SEM, n = 10 in each group. ^##^*P* < 0.01 compared with the control group. ^*^P < 0.05, **P < 0.01 compared with the PQ group.

After 56 h of PQ injection, the serum CRP, IL-6, NF-κB levels all signiﬁcantly increased in PQ group compared with control (Figure 4). In all of the combination treatment groups (SF+OMT 1.6 + 3.4, 3.1+6.9 and 6.2 + 13.8 mg/Kg/day), the level of IL-6 in serum was significantly decreased in a dose-dependent manner (*P* < 0.05 in the low dose group; *P* < 0.01 in the middle dose group and high dose group). The levels of CRP and NF-κB in serum significantly decreased in the middle dose group and high dose group of the combination treatment (*P* < 0.01). Except the level of IL-6 in serum decreased significantly (*P* < 0.05) in OMT (6.9 mg/Kg/day) group, other measured index did not exhibit significant change in SF or OMT alone treatment group.

*Effect* *s* *of SF and OMT combination on the levels of CRP, IL-6 and NF-* *κ* *B in lung homogenate of rat induced by PQ* *of* *rat* *s*

**Figure 5:**
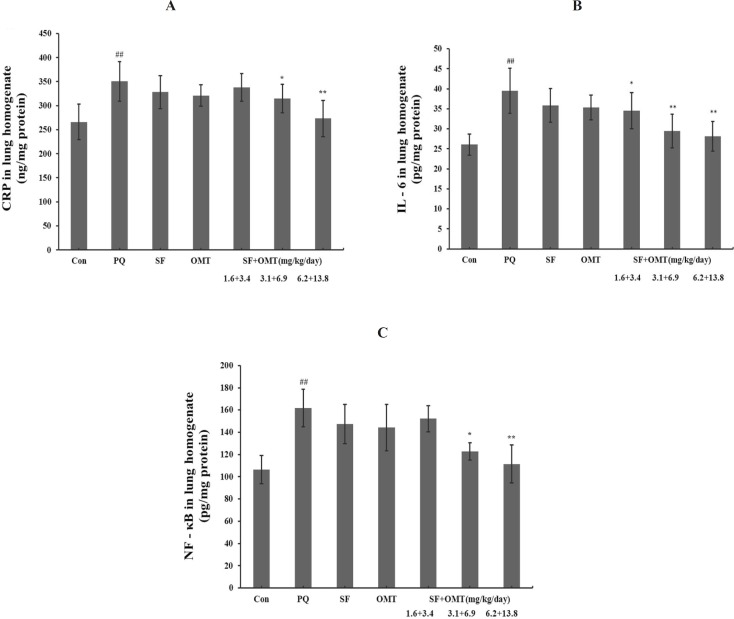
Effects of SF and OMT combination on the levels of CRP (a), IL-6 (b), NF-κB (c) in the lung homogenate of the PQ-intoxication rats. CON, PQ, SF and OMT represent the control group, paraquat group, sodium ferulate group (3.1 mg/Kg/day) and oxymatrine group (6.9 mg/Kg/day), respectively. SF+OMT represents sodium ferulate and oxymatrine combination treatment groups, and the 1.6 + 3.4, 3.1 + 6.9 and 6.2 + 13.8 respectively represent drug administration doses of SF + OMT in the low dose group, middle dose group and high dose group. Data are expressed as means ± SEM, n = 10 in each group. ^##^*P* < 0.01 compared with the control group. ^*^P < 0.05, **P < 0.01 compared with the PQ group.

As shown in Figure 5, after PQ injection, the lung homogenate CRP, IL-6 and NF-κB levels significantly increased in PQ group compared with the control. Compared with the PQ group, the levels of CRP, IL-6 and NF-κB signiﬁcantly decreased in the middle dose group and high dose group of SF and OMT combination treatment. In the low dose group of combination treatment (SF+OMT 1.6 + 3.4 mg/Kg/day), only the level of IL-6 in lung homogenate decreased significantly (*P* < 0.05). Compared with PQ group, treatment with SF (3.1 mg/Kg/day) or OMT (6.9 mg/Kg/day) alone could not significantly decrease the levels of CRP, IL-6 and NF-κB in lung homogenate.

*Effects* *of SF and OMT combination on MDA level* *and SOD activity in serum and lung homogenate of rats*

**Figure 6:**
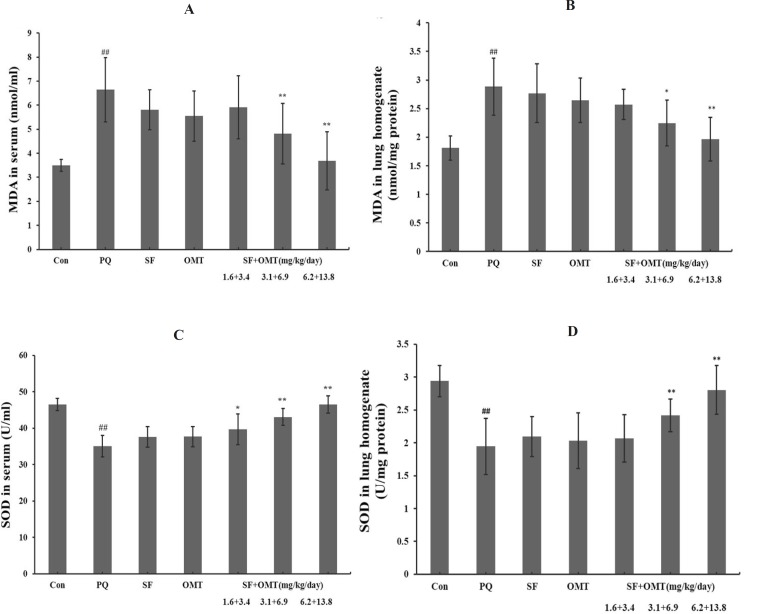
Effects of SF and OMT combination on MDA levels and SOD activities of the PQ-intoxication rats. a: MDA levels in the serum; b: MDA levels in the lung homogenate; c: SOD activities in the serum; d: SOD activities in the lung homogenate. CON, PQ, SF and OMT represent the control group, paraquat group, sodium ferulate group (3.1 mg/Kg/day) and oxymatrine group (6.9 mg/Kg/day), respectively. SF+OMT represents sodium ferulate and oxymatrine combination treatment groups, and the 1.6 + 3.4, 3.1 + 6.9 and 6.2 + 13.8 respectively represent drug administration doses of SF+OMT in the low dose group, middle dose group and high dose group. Data are expressed as means ± SEM. n = 10 in each group. ^##^*P* < 0.01 compared with the control group. ^*^P < 0.05, **P < 0.01 compared with the PQ group.

As shown in Figure 6, a remarkable increase in MDA levels and decrease in SOD activities in serum and lung homogenate were observed in PQ group. Compared with the PQ group, in the combination treatment groups, the level of MDA significantly decreased in serum and lung homogenate with a dose-dependent manner, and the SOD activity significantly increased in serum and in lung homogenate. In the group of treatment with SF (3.1 mg/Kg/day) or OMT (6.9 mg/Kg/day) alone, the MDA level and SOD activity in serum and in lung homogenate did not exhibit significant efficacy.

## Discussion

In recent years, it had become a popular research topic to find a better therapeutic efﬁcacy of multiple drugs combination. Many academics had been involved in this field. As a compound extracted from Radix Angelicae sinensis (named Danggui in Chinese), sodium ferulate (SF) had been used to treat cerebrovascular and cardiovascular diseases because of its effects on anti-inﬂammation, antioxidant and platelet aggregation inhibition (16, 17). As the active component of Chinese herb Radix Sophora ﬂavescens Ait (named Kushen in Chinese), OMT had been reported with the following pharmacological effects, anti-inﬂammation, anti-apoptotic, protecting hepatocytes, and inhibiting immune reaction (18). The traditional Chinese recipe composed of Danggui and Kushen had been used to treat inﬂammation diseases for many years. Such as the Danggui Kushen pill was used to treat the acne of youth and the effects were accepted by the doctors and the patients. In China, there were many reports about the anti-inflammatory effect of SF or OMT alone. But the experimental results confirmed that its anti-inflammatory effect was not strong, so SF or OMT alone was not used in the clinic as anti-inflammatory drugs. In our previous studies, the synergistic anti-inﬂammatory and antioxidant effects of the SF and OMT combination had been demonstrated respectively on the xylene-induced ear edema (10), LPS induced pulmonary edema (15) and sepsis induced lung injury (19) of mice. So, we used SF and OMT combination to treat the PQ-induced lung injury and hope to find an effective drug for the PQ intoxication treatment.

The rodents intraperitoneal injection PQ model had been extensively used to study the intervention of PQ induced lung injury. In this study, we duplicated the PQ induced acute death model in mice, in which the animals began to die at about 40 hours after PQ injection, and died to 100% at about 52 h, but the lung injury was not obvious and the lung W/D ratio was not changed significantly (data not shown). Those results suggested us that Swiss mouse was not a good animal model to duplicate the PQ-induced acute lung injury and the main injured target organ which lead to the animals death need to be explored. In rat PQ intoxication model, the lung injury was markedly reflected by dramatically increased lung W/D ratio, lung edema, hemorrhage and histopathological scores. So the rat PQ intoxication model was fit for observation of the drug treatment efficacy on the PQ induced lung injury. In our study, we administered the drug 30 min after PQ injection with hypodermic injection, which could mimic the time of patient transferring to the hospital after PQ intoxication. In addition, it was considered that the drug was absorbed slower with hypodermic injection than the venous and intraperitoneal injection. So, the hypodermic injection method was selected to administer the drug, through which the speed of drug absorption was closer to venous drop injection in clinic. The results indicated that the PQ induced mice mortality decreased, the death time prolonged, the increased rat lung W/D ratio and lung histopathological score all attenuated after treatment with SF and OMT combination, which exhibited protective effect on PQ injury.

It was thought that anti-inflammatory effects of SF and OMT combination might be involved in the protective mechanism on PQ induce injury. In our previous study (10), we found that SF and OMT combination could synergistically down-regulated IL-6, CRP genes in LPS-stimulated RAW 264.7 cells. So, the inflammatory cytokine included CRP, IL-6, NF-κB levels were measured after 56 hours of PQ injection when there was no animals died, and the detected index all increased markedly.

CRP, as an indicator of systemic inﬂammatory reaction, was a kind of reactive protein in acute inflammation phrase (20), which exhibited pro-inﬂammatory effects by inducing inﬂammatory cytokines and played key role on activating the complement system and tissue factor production in monocytes (21). CRP level showed a significant positive correlation with tissue damage. In recent years, it was considered that CRP could predict the risk of the occurrence of organ damage and was known as a marker of inflammation reaction (22). IL-6, as a pro-inflammatory cytokine, had many biological features and played an important role in the process of inflammation (23). It was reported that IL-6 level was greatly up-regulated with PQ-induced lung injury (24). NF-κB, as an indicator of systemic inﬂammatory reaction, could induce inﬂammatory protein production, activate the gene transcription system and tissue factor production in nuclei pulp. As the results showed, treatment with SF and OMT combination signiﬁcantly attenuated the PQ increased CRP, IL-6, NF-κB levels, which related to the acute phase of the inflammatory response. These results suggested us that the anti-inflammatory effect of SF and OMT combination involved in the protective mechanism, and further certified the anti-inflammatory effect of SF and OMT combination. Otherwise, this was the first report for the effect of SF and OMT combination on the NF-κB and the reaction pathway would be studied further in our future studies.

One of histopathological reason of PQ-induced lung injury was that too much reactive oxygen species was produced. That the intraperitoneal injection of PQ induced the generation of superoxide anions, hydroxyl radicals, and oxidant peroxynitrite which led to oxidative stress damage-related cell death and inﬂammation was demonstrated in many reports (2, 7, 25). MDA was one of the major secondary oxidation products derived from polyunsaturated fatty acids. As an index of structural oxidative injury of cell membrane, MDA was a well-known parameter for determining the increased free radical formation. An increase in MDA level suggested increased lipid peroxidation initiated by free radical reactions (13). SOD, as an antioxidant enzyme, had a capable of scavenging the superoxide free radical and confronting the cell damage from the free radical. The enhancement of oxidizing reaction was reﬂected by decrease SOD activity. In our study, the results showed that the MDA levels in serum and lung tissue were dramatically increased by PQ injection and the SOD activity was dramatically decreased. However, this increased MDA level induced by PQ injection was signiﬁcantly decreased by SF and OMT combination treatment, and this SOD activity decrease was signiﬁcantly improved. So that, it suggested that oxidative damage in serum and in the lung tissue were alleviated after the treatment with SF and OMT combination.

In order to further validate the synergistic effects of SF and OMT combination, we designed other two groups which were treated with the SF or OMT alone. Because the markedly protective effect was observed in middle dose group of the combination, we designed the dose of the drug SF or OMT alone was 3.1 mg/Kg/day and 6.9 mg/Kg/day which corresponded to middle dose group of the combination treatment (SF +OMT 3.1 + 6.9 mg/Kg/day). The results show that, compared with the PQ group, no obvious antioxidant or anti-inflammatory effects were observed in the SF (3.1 mg/Kg/day) or OMT (6.9 mg/Kg/day) group except the serum IL-6 level decreased in OMT (6.9 mg/Kg/day) group. The results indirectly confirmed the synergistic effect of the combination of those two drugs, and were accordance with our previous report (10, 15, 26).

## Conclusions

SF and OMT combination had an obvious protective effect on PQ-induced lung injury. The anti-inﬂammatory and antioxidant effect might be involved in the mechanism.
